# B-virus from pet macaque monkeys: an emerging threat in the United States?

**DOI:** 10.3201/eid0401.980117

**Published:** 1998

**Authors:** S R Ostrowski, M J Leslie, T Parrott, S Abelt, P E Piercy

**Affiliations:** Centers for Disease Control and Prevention, Atlanta, Georgia, USA.

## Abstract

Of primary concern when evaluating macaque bites are bacterial and B-virus infections. B-virus infection is highly prevalent (80% to 90%) in adult macaques and may cause a potentially fatal meningoencephalitis in humans. We examined seven nonoccupational exposure incidents involving 24 persons and eight macaques. Six macaques were tested for herpes B; four (67%) were seropositive. A common observation was that children were more than three times as likely to be bitten than adults. The virus must be assumed to be a potential health hazard in macaque bite wounds; this risk makes macaques unsuitable as pets.


### B-virus from Pet Macaque Monkeys: An Emerging Threat in the United States?

Of primary concern when evaluating macaque bites are bacterial and B-virus infections. B-virus infection is highly prevalent (80% to 90%) in adult macaques and may cause a potentially fatal meningoencephalitis in humans. We examined seven nonoccupational exposure incidents involving 24 persons and eight macaques. Six macaques were tested for herpes B; four (67%) were seropositive. A common observation was that children were more than three times as likely to be bitten than adults. The virus must be assumed to be a potential health hazard in macaque bite wounds; this risk makes macaques unsuitable as pets.

### B-Virus in Nonhuman Primates

*Cercopithecine herpesvirus 1* (*Herpesvirus simiae* or B-virus) frequently infects Old World primates of the genus *Macaca*. Of at least 19 species of macaques, rhesus, Japanese, cynomolgus, pig-tailed, and stump-tailed macaques are the species most commonly used in biomedical research (1). Seroprevalence of neutralizing antibodies to B-virus in captive adult macaque populations is 73% to 100% (1-3). Like *Herpesvirus simplex* virus infection in humans, B-virus infection in monkeys is characterized by lifelong infection with intermittent reactivation and shedding of the virus in saliva or genital secretions, particularly during periods of stress or immunosuppression (4). B-virus infection is transmitted among free-ranging or group-housed animals, primarily through sexual activity and bites. In captivity, as well as in the wild, mature macaques are more likely than immature animals to have been infected with, and shed, the virus. Antibody titer to B-virus indicates infection but can neither confirm nor eliminate actual viral shedding at the time of the bite (4).

### B-Virus in Humans

B-virus disease in humans usually results from macaque bites or scratches (4). Incubation periods may be as short as 2 days, but more commonly are 2 to 5 weeks (1,3,5-7; Centers for Disease Control and Prevention [CDC], unpub. data). Most documented infections have occurred among biomedical research employees who had occupational exposure to macaques, although transmission has also been documented among laboratory workers handling infected central nervous system and kidney tissues (1,5).

From 1990 to 1992, 28 U.S. residents reported nonoccupational macaque bites to CDC (L. Chapman, pers. comm.). Since 1993, additional nonoccupational exposure cases have been reported, seven of which (involving 24 persons and eight macaques) are listed in Table 1. Of the six macaques for which herpes B serologic results were available, four (67%) were positive. Two owners refused requests for testing. Four (44%) of nine exposed children were bitten, versus only three (20%) of 12 adults. Children were 3.2 times more likely to be bitten than adults; although a common observation, this association is not statistically significant for this case series.

Most free-ranging monkey populations are thought to be part of the exotic fauna of distant tourist destinations and wild animal parks; however, macaque species have established free-ranging feral populations in Texas and Florida. In such settings, contact between humans and macaques cannot be safely controlled (8-10), and workers and visitors are at risk. Guidelines for B-virus prevention and diagnosis have recently been published (9-12).

Symptomatic human infection with B-virus is rare; fewer than 40 cases were reported from 1933 to 1994 (1,4-7,13-15; CDC, unpub. data). However, the consequences of symptomatic infection may be severe. Viral infection rapidly progresses to central loci in the spinal cord and, eventually, the brain. Of 24 known symptomatic patients whose cases were reviewed in 1992, 19 (79%) died (CDC; unpub. data).

Before 1987, most surviving human patients had moderate to severe neurologic impairment, sometimes requiring lifelong institutionaliza-

Table 1. Selected pet macaque bite cases[a,b]

| Location | Primate species, age, B-virus status | Nature of exposure | Comments |
|---|---|---|---|
| Illinois | Rhesus, 20+ yrs, B-virus positive Cynomolgus, 2-4 yrs, B-virus negative | Household contact (2 adults/3 children), bites, scratches (2 adults) | Bought at auction, wife bitten multiple sites, children hand-fed monkey |
| Florida | Cynomolgus, 2 yrs, B-virus positive | Household contact (1 adult), bite (1 child) | Kissed on lips, ate off owner's plate, shared bed |
| Arizona | Cynomolgus, 2 yrs, B-virus negative | Bites on toe and buttock (child) | Unprovoked attack on neighbor, declared vicious animal by judge, no. of household contacts (owner) unreported |
| | Cynomolgus, 7 weeks, B-virus positive | Household contact (6 adults), bite on face (1 adult) | Diapered, shared chewed gum, oral ulcers noted by veterinarian, bite incident at neighborhood bar |
| | Macaque, (species undetermined), 2 yrs, B-virus status unknown | Bite on thigh (1 child) | Unprovoked attack (climbed fence to bite child) |
| | Macaque, (species and age undetermined), B-virus status unknown | Severe bite (1 child) | Injured child attended an unlicensed day-care facility run by monkey owner, 7 other monkeys on premises |
| Minnesota | Rhesus, 2 yrs, B-virus positive | Household contact, owners' friend bitten | Acquired as "child-substitute" (full-time baby-sitters hired) |

[a]Cases referred to Centers for Disease Control and Prevention since 1993.
[b]As of November 1997, no confirmed transmission of B-virus in these persons has been documented.

tion (1). Recently, acyclovir has prevented progression of the disease in a limited number of patients. In at least three patients, this treatment reversed the neurologic symptoms and was life-saving (7,14,15). Rapid diagnosis and initiation of therapy are of paramount importance in preventing death or permanent disability in surviving patients.

### Human and Macaque Interactions

Most owners form an emotional bond with infant primates. This bond is probably strengthened by the neonatal monkey's physical and behavioral resemblance to a human infant. Although physically and emotionally dependent on their mothers (or human substitutes) for up to 2 years of age, most macaques exhibit unpredictable behavior as they mature. Males tend to become aggressive, and both male and female macaques bite to defend themselves and to establish dominance. Dominance within the social hierarchy of macaques is established by aggression toward other monkeys, generally the younger and smaller members of the group. Both veterinary specialists and breeders of nonhuman primates agree that as a rule, all these animals bite (16,17). Biting incidents eventually bring the animals to the attention of animal control authorities. Most state health departments can require that any biting nondomestic animal be euthanized and the brain be submitted for rabies testing.

### Regulations, Guidelines, and Policies Regarding Nonhuman Primates

Table 2 lists the principal federal regulations affecting the possession, distribution, and uses of nonhuman primates. The United States is obligated under the Convention in International Trade in Endangered Species (CITES) to restrict and control trafficking in exotic and endangered species.

Since October 10, 1975, U.S. Public Health regulation 42 CFR 71.53(c) has prohibited the importation of nonhuman primates into the United States as pets, and neither nonhuman primates imported since that date nor their offspring may be legally bred or distributed for any uses other than bona fide science, university-

Table 2. Federal regulations regarding nonhuman primates

| Agency | Statute | Regulation | Subjects |
|---|---|---|---|
| Departments of Health, Centers for Disease Control and Prevention | Public Health Services Act, 42 USCS 201 | 42 CFR 71.53 | Importation, distribution, bona fide uses in the U.S., breeding colony requirements, pet/avocationist uses |
| Department of Agriculture | Animal Welfare Act, 7 USCS 2131-2159 | 9 CFR Subchapter A | Licenses (breeders, dealers, laboratories, exhibitors, auctions), interstate health certificates, humane care and transport |
| Department of the Interior, U.S. Fish and Wildlife Service | Endangered Species Act, 16 USCS 1540 Lacey Act, 18 USCS 42 | 50 CFR 10, 11, 13, 14, 16 | Endangered species, smuggling, interstate sales |

level educational programs, or full-time zoologic exhibition. Furthermore, the regulation states, “the maintenance of nonhuman primates as pets, hobby, or an avocation with occasional display to others is not a permissible use” (18).

All states require their citizens to comply with applicable federal regulations. Many state officials, however, may be unaware of regulatory restrictions on the uses and distribution of nonhuman primates and may be confused by the distinctions among federal agencies regarding regulatory restrictions on captive-bred animals. State wildlife authorities may not know that a federal public health regulation prohibits the keeping (“maintenance”) of nonhuman primates imported after October 10, 1975, as pets, for a hobby, or as an avocation; likewise, many do not know the compelling public health and safety reasons for enforcement.

Captive-bred offspring of animals purported to have been imported before October 10, 1975, are frequently offered for sale. Without documentation it is very difficult to determine whether this is the case. Depending on the specific circumstances, it is possible for undocumented animals to be considered deliberately misclassified (i.e., intentionally mislabeled), a violation under the Lacey Act (18 USC 42) and under 16 USC 3373 (19).

In 1987 and 1988, occupational safety guidelines were published based on evidence that all macaque species are inherently dangerous to humans because of the risk for B-virus transmission, as well as the likelihood of serious physical injury from bite wounds (9-12,14,15). Several recent reviews of monkey-bite injuries worldwide indicate that severe lacerations, wound infections, and permanent sequelae (e.g., flexure contractures, osteomyelitis) were present in 33% of cases (20,21).

In 1990, the American Veterinary Medical Association issued a general policy statement opposing the keeping of wild animals (especially those inherently dangerous to humans) as pets and advising veterinarians to exert their influence to discourage this practice (22). In 1995, updated guidelines for the prevention and treatment of B-virus infections in exposed persons were published (12). Despite these continuing public health educational efforts, nonhuman primates (including macaques) continue to be marketed and kept as pets in many states (16,17,23).

### The Frequency of Exposure Resulting in Infection

Much remains to be learned about the pathogenesis of B-virus infection in humans. In this very limited case series (Table 1), one family (two adults and two of three children) exposed to a B-virus positive macaque had flulike symptoms. One of the adults had additional symptoms related to the injury site, which suggested B-virus infection. In the other six cases, no suspect clinical symptoms were noted, and disease-specific antiviral postexposure prophylaxis was not given. B-virus is still rare, and diagnostic evaluation of clinical cases of aseptic meningitis does not routinely include B-virus testing.

Owners of pet macaques are often reluctant to report bite injuries from their pets, even to their medical care providers, and may fail to appreciate that the premonitory headache and flulike symptoms (which may lead them to seek medical attention) could be associated with healed, often minor, bite wounds dating back more than a month (23). The Southwest Foundation for

Biomedical Research, which is the designated National Institutes of Health B-virus resource laboratory, reports processing 2,000 to 3,000 human diagnostic specimens per year between 1990 and 1994, or approximately 200 per month, most of which reflect occupational exposure (8).

### Some Public Health Consequences of the Nonhuman Primate Pet Trade

The pet trade in a variety of nonhuman primate species, and particularly the apparent increase in macaque species as part of this trade, may constitute an emerging infectious disease threat in the United States. Although the U.S. Fish and Wildlife Service indicates that illegal traffic in nonhuman primates is a significant aspect of the estimated $3 billion worth of wildlife illegally traded in the United States annually, more data are needed on the actual number of macaques in the private sector and on trends in the population (24; U.S. Fish and Wildlife special agents, pers. comm.).

The public resources deployed when a monkey-bite case is referred to public health authorities are similar to those required for rabies investigations (M. Leslie and T. Parrott, unpub. obs.). Persons bitten by pet and feral macaques are more likely than persons bitten in the workplace to require public resources, delay seeking medical care, and have an initial medical evaluation by care givers who are largely unfamiliar with the potentially serious consequences of B-virus exposure (23). In contrast, occupational exposure generally occurs within highly structured workplace settings, where health professionals are prepared to provide prompt, appropriate, and specific care at no public cost.

Ongoing efforts to establish B-virus–free macaque colonies illustrate the difficulties of ascertaining B-virus–negative status, even with a battery of sophisticated laboratory tests and extended longitudinal follow-up of individual macaques (25). The high percentage of death in known cases of human B-virus disease underscores the potential seriousness of all bite or scratch exposures from macaques.

The extremely high prevalence of B-virus along with their behavioral characteristics make the macaque species unsuitable as pets.

### Acknowledgments

The authors thank Drs. D. Manning, S. O' Marro, R. Montrey, T. Burke, D. Morton, and J. Thulin; Mr. J. Francis, Mr. C. Langkop; Drs. R. Martin, J. Hilliard, D. Watkins; Capt. J. Thompson; Special Agents P. Bosco, D. Burleson, J. English, S. Hamilton, T. Karabinoff, D. Kirkby, D. Manera, G. Phillips, G. Phocas, T. Santelle, J. Sommers, C. Tabor; Dr. J. Cheek; and the Division of Viral and Rickettsial Diseases, Animal Resources Branch, and the Division of Quarantine, CDC.

**Stephanie R. Ostrowski,* Mira J. Leslie,† Terri Parrott,‡ Susan Abelt,¶ and Patrick E. Piercy§**

*Centers for Disease Control and Prevention, Atlanta, Georgia, USA; †Arizona Department of Health Services, Phoenix, Arizona, USA; ‡Pembroke Park Animal Hospital, Pembroke Park, Florida, USA; ¶Lake Superior Zoological Gardens, Duluth, Minnesota, USA; and §Illinois Department of Public Health, Springfield, Illinois, USA

### References

1. Palmer AE. B virus, *Herpesvirus simiae*: historical perspective. J Med Primatol 1987;16:99-130.
2. Shah KV, Morrison JA. Comparison of three rhesus groups for antibody patterns to some viruses: absence of active simian virus 40 transmission in the free-ranging rhesus of Cayo Santiago. Am J Epidemiol 1972;99:308-15.
3. Orcutt RP, Pucak GJ, Foster HL, Kilcourse JY. Multiple testing for the detection of B virus antibody in specially handled rhesus monkeys after capture from virgin trapping grounds. Lab Anim Sci 1976;26:70-4.
4. Weigler BJ. Biology of B Virus in macaque and human hosts: review. Clin Infect Dis 1992;14:555-67.
5. Hummeler K, Davidson WL, Henle W, LaBoccetta AC, Ruch HG. Encephalomyelitis due to infection with *Herpesvirus simiae* (Herpes B virus): report of two fatal laboratory acquired cases. N Engl J Med 1959;261:64-8.
6. Centers for Disease Control. B-virus infections in humans—Michigan. MMWR Morb Mortal Wkly Rep 1989;38:453-4.
7. Holmes GP, Hilliard JK, Klontz KC, Rupert AH, Schindler CM, Parrish E, et al. B-virus (*Herpesvirus simiae*) infection in humans: epidemiologic investigations of a cluster. Ann Intern Med 1990;112:833-9.
8. Centers for Disease Control. Guidelines for prevention of *Herpesvirus simiae* (B-virus) infection in monkey handlers. MMWR Morb Mortal Wkly Rep 1987;36:681-2, 687-9.
9. The B Virus working group. Guidelines from prevention of *Herpesvirus simiae* (B virus) infection in monkey handlers. J Med Primatol 1988;17:77-83.
10. Hilliard JK. 1990-1994 yearly comparisons; B-virus Resource Laboratory. San Antonio (TX): Southwest Foundation for Biomedical Research; 1995.
11. Wells DL, Lipper SL, Hilliard JK, Stewart JA, Holmes GP, Herrmann KL, et al. *Herpesvirus simiae* contamination of primary rhesus monkey kidney cell cultures. CDC recommendations to minimize risks to laboratory personnel. Diagn Microbiol Infect Dis 1989;12:333-6.

12. Holmes GP, Chapman LE, Stewart JA, Straus SE, Hilliard JK, Davenport DS, et al. Guidelines for the prevention and treatment of B-virus infections in exposed persons. Clin Infect Dis 1995;20:421-39.
13. Artenstein AW, Hicks CB, Goodwin BS, Hilliard JK. Human infection with B virus following a needlestick injury. Reviews of Infectious Diseases 1991;13:288-91.
14. Centers for Disease Control. B-virus infection in humans—Pensacola, Florida. MMWR Morb Mortal Wkly Rep 1987;36:289-90, 295-6.
15. Centers for Disease Control. B-virus infection in humans—Michigan. MMWR Morb Mortal Wkly Rep 1989;38:453-4.
16. Hoctor P. Short hints on raising exotics. Animal Finders Guide 1995 Sep 1;12:44-5.
17. Johnson-Delaney CA. The pet monkey: health care and husbandry guidelines. Journal of Small Exotic Animal Medicine 1991;1:32-7.
18. Centers for Disease Control. Import restrictions on nonhuman primates. Atlanta (GA): Centers for Disease Control; 1982 Nov 17. DQ-CPS Advisory Memorandum No. 65.
19. Anderson RS. Lacey Act. Case law update and current issues in federal wildlife prosecution. Washington: U.S. Department of Justice, Environment and Natural Resources Division; 1993.
20. Goldstein EJC, Pryor EP, Citron DM. Simian bites and bacterial infection. Clin Infect Dis 1995;20:1551-2.
21. Janda DH, Ringler DH, Hilliard JK, Hankin RC, Hankin FM. Nonhuman primate bites. J Orthop Res 1990;8:146-50.
22. American Veterinary Medical Association. Policy Statement on Wild Animals as Pets. In: 1995 AVMA Directory. Schaumburg (IL): The Association; 1995.
23. Paulette J. “Yes, monkeys will bite!” The Simian 1996 Feb;6-8.
24. Webster D. The looting and smuggling and fencing and hoarding of impossibly precious, feathered and scaly wild things: inside the $10 billion black market in endangered animals. The New York Times Magazine 1997 Feb 16: 27-33, 48-50.
25. Hilliard J. Managing macaques and herpes B. Presented at 4th National Symposium on Biosafety: Working Safely with Research Animals; 1997 Jan 27-31; Atlanta, Georgia.
